# Aneurysm and Aortic Insufficiency

**Published:** 1918-09

**Authors:** 


**Aneurysm and Aortic Insufficiency.** Felix Ramond and Postina, Le Prog. Med., Mch. 9, report a case in a young soldier, aortic dilatation and insufficency of the valve following a wound from an aerial torpedo. Death ensued in three months (rupture not being mentioned). Necropsy revealed that the essential cause was an old syphilitic aortitis, the traumatism being an added factor.

